# Under pressure - Nursing staff's perspectives on working hours and recovery during the COVID-19 pandemic: A qualitative study

**DOI:** 10.1016/j.ijnsa.2024.100225

**Published:** 2024-07-15

**Authors:** Isabelle Hernandez, Marie Söderström, Ann Rudman, Anna Dahlgren

**Affiliations:** a Department of Clinical Neuroscience, Karolinska Institutet, Solna, Sweden; b School of Health and Welfare, Dalarna University, Falun, Sweden

**Keywords:** COVID-19, Fatigue, Occupational stress, Nursing, Personnel management, Shift work schedule, Sleep, Work-Life balance

## Abstract

**Background:**

The COVID-19 pandemic contributed to increased pressure on healthcare systems. During periods when the demands exceed the capacity of healthcare organizations, adaptive strategies are used to meet these demands. During the COVID-19 pandemic, working hours for nursing staff were reorganized and extended. This has posed challenges for recovery, which may be a key factor for maintaining health and safety under such conditions.

**Objectives:**

The aim of the study was to bring insights into how nursing staff perceived their working hours and recovery during the COVID-19 pandemic, and if they experienced any changes in their sleep and well-being.

**Design:**

A qualitative descriptive design was chosen, as it is suitable for gaining insight into perceptions and experiences.

**Methods:**

Qualitative semi-structured interviews were conducted using an interview guide. The interviews were analyzed using thematic analysis. Sixteen registered nurses and six certified nursing assistants from four Swedish hospitals participated in the study.

**Results:**

The organization of working hours during the COVID-19 pandemic was considered suboptimal and resulted in more demanding working hours and poor recovery. Nursing staff experienced loss of control as they lost influence over working hours, working hours became more unpredictable and the boundaries between work and leisure became blurred. Nursing staff also experienced a decline in their health and well-being, including extreme fatigue, impaired sleep and physical/mental changes.

**Conclusion:**

The strategies used by healthcare organizations to meet increasing demands during the COVID-19 pandemic contributed to impaired recovery and well-being of nursing staff, which could generate negative feedback loops contributing to depletion of resources at the organizational level.

## Abstract (author highlights)

What is already known

• Overtime, long working hours and night work have been associated with health and safety risks.
• Organizations can flex their human resources by extending working hours when under pressure.
• Recovery may be a key factor for preventing adverse consequences of demanding working hours and job stressors.

**What this paper adds**

• Suboptimal organization of working hours during periods of increased pressure can lead to loss of control and negatively impact sleep and well-being among employees.
• Lost control over working hours and free time can contribute to poor recovery and ill-health.
• Short-term organizational-level strategies used during the COVID-19 pandemic contributed to negative consequences at the employee-level, which could have a wider system impact.

## 1 Background

Since the outbreak of the pandemic in early 2020, over 770 million confirmed cases of COVID-19 have been reported worldwide (World Health Organization, 2023), and the healthcare sector has faced major challenges in adapting to the increasing demands during this period. Qualitative findings from several countries suggest that nurses have been subjected to stressors such as intense workload, fear of contracting the virus, and having to wear heavy protection gear during work shifts (Escher et al., 2023; Goh et al., 2021; Popoola et al., 2022). A comprehensive amount of literature has highlighted that the pandemic resulted in a high prevalence of stress-related illness among healthcare workers, including anxiety, depression, insomnia, burnout and posttraumatic stress (Chutiyami et al., 2022).

Review findings suggest that demanding working hours could be one contributing factor to both negative mental health outcomes (Britt et al., 2021; Umbetkulova et al., 2024), and turnover intention (Poon et al., 2022) among healthcare workers during the pandemic. Long working hours have for example been associated with higher levels of stress, burnout and intention to leave the job amongst nurses (Hoedl et al., 2021; Li et al., 2022). Working >40 h per week has been associated with reporting higher chronic and acute fatigue, and poorer intershift recovery among nursing staff in the United States (Sagherian et al., 2023). Qualitative findings suggest that long working hours and poor work-life balance were among the specific challenges experienced by nurses during the COVID-19 pandemic (Sperling, 2021), and working long (<12 h) shifts while caring for COVID-19 patients has been considered challenging (Goh et al., 2021). It has also been found that working fixed night shifts during the pandemic was related to significantly higher occupational stress amongst nurses, compared to working fixed day or rotating shifts (Şanlıtürk, 2021).

Shift work is prevalent in 24/7-hour operations such as healthcare and has in general been linked to poor health outcomes (Kecklund and Axelsson, 2016). Research conducted prior to the pandemic demonstrates that long working hours (i.e. >10–12 h shifts, long work weeks, overtime) are associated with adverse health outcomes, poor sleep (Wong et al., 2019), increased risk of occupational accidents and injuries (Fischer et al., 2017; Wagstaff and Lie, 2011) and sickness absence (Dall'Ora et al., 2018). Other scheduling characteristics such as irregular working hours and short shift intervals (<11–13 h between shifts) have also been associated with increased sickness absence (Dall'Ora et al., 2018; Rosenström et al., 2021). A potential buffer against negative health consequences due to demanding working hours is employee influence over work schedules (Ala-Mursula et al., 2005). In the Swedish healthcare sector, employees are often given such influence trough participatory working time scheduling (Epstein et al., 2023). However, during periods when healthcare organizations are under increased pressure, it is unclear whether participatory working time scheduling is beneficial.

Impaired sleep, circadian disruption, and lack of recovery are potentially important mechanisms behind the associations between shiftwork, demanding working hours and negative health outcomes (Kecklund and Axelsson, 2016; Geurts and Sonnentag, 2006). Recovery is a restorative process involving sleep and recovery activities, and a possible key factor for preventing stress-related ill-health (Geurts and Sonnentag, 2006). Plausibly, it is even more important to get sufficient recovery during periods of high stress. However, it has been suggested that recovery can be disrupted under these periods, as high stress can for example impair sleep and lead to difficulties to psychologically detach from work during free time. This phenomenon has been referred to as the *recovery paradox* (Sonnentag, 2018). Research findings indicate that the recovery of healthcare workers was impaired during COVID-19 pandemic (Jonsdottir et al., 2021; Marvaldi et al., 2021). Shift work together with intense workload and (or) extended working hours, as during the pandemic, could possibly lead to even more pronounced negative health or safety outcomes.

Page and colleagues (2023) have developed a taxonomy of adaptive strategies utilized by healthcare organizations in situations when the demands exceed the capacity of organizations, such as during the COVID-19 pandemic. Flexing organizational resources by for example extending working hours is one such strategy. While these strategies serve an important purpose in responding to changing circumstances, they may also lead to negative consequences on both the individual and the organizational level, referred to as negative feedback loops, which in turn can aggravate the imbalance between organizational demands and capacity (Page et al., 2023). Accordingly, qualitative findings show that during the pandemic, Swedish healthcare organizations tried to meet the increased demands by maximizing working hours (e.g. increasing working hours, implementing new schedules, limiting vacation) and reorganizing employees, care processes and HR (e.g. redeploying staff, pausing surgeries) (Hernandez et al., in press), suggesting that substantial changes in the daily operations were made to meet increasing demands during the pandemic. While these strategies may be beneficial to handle the situation short-term, they could have long term consequences for organizations by depleting their human capital through e.g. sick leave and turnover. This is referred to as an *organizational recovery paradox* (Hernandez et al., in press).

While previous studies have highlighted the negative effects of demanding working hours during the pandemic, little is known about healthcare workers own experiences and descriptions of their working hours and recovery during the pandemic, a period during which the healthcare organizations used short-term solutions and implemented several changes in their daily operations in order to handle the increased pressure. Thus, the aim of the present study was to bring insights into how nursing staff perceived their working hours and recovery during the COVID-19 pandemic, and if they experienced any changes in their sleep and well-being.

## 2 Methods

### 2.1 Design

A qualitative descriptive design was chosen, as it is suitable for gaining insight into perceptions and experiences of the participants through their descriptions of the phenomenon under study (Sandelowski, 2000). The following inclusion criteria was set; 1. being a registered nurse or a certified nursing assistant, 2. holding a permanent job position and 3. having worked with COVID-19 patients during the pandemic.

### 2.2 Context

During the pandemic, working hours for healthcare staff were managed differently between the Swedish regions and during different periods of the pandemic. One approach, used in some regions, was to go from participatory working time scheduling to fixed schedules. Other regions utilized the crisis agreement which increased the weekly working hours from 40 to 48 h and extended the shift lengths to 12,5 h. Some participants in the present study had fixed schedules during a six-month period which followed a consecutive pattern of four workdays followed by two days off, referred to as 4–2 schedules. For further details on the different types of scheduling used in Swedish 24/7 healthcare during the pandemic, see Hernandez et al. (in press).

### 2.3 Participants

Participants were nurses and certified nursing assistants (all referred to as “nurses”) who were chosen through convenience sampling in four hospitals in three Swedish regions. Participants from different regions were chosen in order to capture a variety of experiences, as strategies for managing working hours had varied between regions during the pandemic (Hernandez et al., in press). Information about the study was distributed via hospital e-mail to employees in relevant work units (e.g. ICU, specific COVID-19 units), as well as through flyers in the workplace with a link/QR-code to a registration site for the study. After stating their interest to participate, participants received an e-mail with further information about the study and research team, as well as a link to sign their informed consent for participation in the study and to provide their contact details. Contact details were used for scheduling the interview and to agree on when and how (telephone/video call) the interview should be conducted. In total, 27 individuals registered for the study, of which five were excluded due to not meeting the inclusion criteria, resulting in 22 included participants.

## 3 Materials

A semi-structured interview guide was developed by the authors and discussed with a couple of stakeholders with insight in working hours within the Swedish healthcare sector prior to the data collection. This was done in order to ensure that no important aspects were missed. A test interview was conducted in November 2020 to verify that the questions were understandable and generated relevant responses. Following the test interview, some adjustments were made to the interview guide before it was finalized. The interview questions are presented in table 1.

**Table 1:** The interview guide.

**Type of question**	**Question**
Background	Could you provide a brief description of your background (age, job role, years of experience, family situation)?
Main questions	How would you, in your own words, describe work during the pandemic? What was the process of scheduling working hours like during the pandemic? What did your working hours look like during the pandemic? Generally, do you experience any difficulties with your sleep, and has it changed during the pandemic? Have you noticed any impact on your health during the pandemic? Can you describe if you have experienced fatigue during the pandemic? Has your employer created possibilities for recovery during the pandemic? What have you done to recover from work during your free time during the pandemic? Has your work affected you during your free time during the pandemic? What do you think are important lessons learned when it comes to the scheduling of working hours and recovery for similar situations in the future?
Follow-up and probing questions	E.g. “Is this different from prior to the pandemic?”, “Please explain more”

### 3.1 Data collection

All interviews were conducted by the first (PhD-candidate), second (PhD, licensed psychologist, researcher) and last (PhD, project leader) author between January 2021 and March 2022. Data were collected via telephone or video call depending on the participants preference, and the participants chose the interview setting (in most cases in their homes). Only the participant and the researcher were present during the interview. The interviews were recorded by audio and lasted between 47 and 131 min (*M* = 77 min). The audio files were transcribed in verbatim and pseudonymized. Throughout the data collection process, the content of the interviews was discussed between the authors, and data collection stopped after 22 interviews when data covered dense and varied descriptions as well as started to repeat itself.

### 3.2 Data analysis

Data was analyzed using thematic analysis following the six steps described by Braun and Clarke (2006; 2022). The first step involved familiarizing with data by listening to recoded audio files, reading through the interview transcripts and taking initial notes. The first author read all interview transcripts, and the second, third (PhD, RN, researcher) and fourth author read several interview transcripts each. The second step involved generating initial codes using an inductive approach looking for information related to the study aim. The entire dataset was coded by the first author and since all authors had familiarized with the data, the coding process could be checked, discussed and confirmed by the other authors. The coding process was carried out directly in the data transcript files and then gathered in a separate document. In step three, which involved developing themes, the first author summarized the content of the interviews based on the codes and the raw data, and all authors independently searched for themes based on their notes, the raw data and the summary of interview contents. The authors then met to discuss their findings and preliminary themes were formed. In step four, the themes were reviewed in several meetings between all authors and in some cases restructured. This included going back to reading raw data to ensure that no important aspects had been missed. In the fifth step of the analysis process, themes were finalized and named, and in the sixth step the results were summarized. An example from the analysis process is illustrated in Table 2. The findings presented in this article are a result of the collaboration between all authors.

**Table 2:** Example from the analysis process.

**Data extract**	**Code**	**Preliminary main theme**	**Final main theme**	**Sub-theme**
You got two day shifts, then a day off, then night shifts, day off, day shifts. But the days in between… Or between the day and the night shifts were equally long. So, when you left your nightshift, you have to sleep all day, so in practice you get shorter time for recovery after a night period than after a period of day-work.	1. Frequent changes between day and night work 2. Short time for recovery after night work	Perspectives on the organization of working hours	Suboptimal organization of working hours	Demanding shifts and shift patterns Little room for recovery

### 3.3 Ethical considerations

This study was approved by The Swedish Ethical Review Authority (dnr. 2020-04230). All participants were included in the study on a voluntary basis, given written information about the study, encouraged to contact a member of the research group if they had any questions and submitted written informed consent prior to data collection. Participants were also informed that they could withdraw their participation in the study without having to give a reason. All results were presented in a way where they were not deducible to any specific individual. Data were handled exclusively by members of the research group.

## 4 Results

Demographic and background characteristics of the participants can be viewed in table 3.

**Table 3:** Demographic and background characteristics of participants.

Characteristics		N (%)
Gender	Female Male	18 (82) 4 (18)
		
Age	25–35 36–45 46–55 55+	4 (18) 6 (27) 7 (32) 5 (23)
		
Family situation	Married Living together with partner Single Children	11 (50) 2 (9) 5 (23) 14 (64)
		
Years of experience in current job role	1–4 5–10 11–20 21–35 35+ Missing	5 (23) 5 (23) 5 (23) 4 (18) 1 (4) 2 (9)
		
Job role	Registered Nurse Certified Nursing Assistant	16 (73) 6 (27)
		
Type of employment	Full-time Part-time Missing	12 (55) 4 (18) 6 (27)
		
Place of employment during the pandemic	Intensive care unit Emergency room COVID-19 unit Infection department Ambulance	13 (60) 3 (14) 4 (18) 1 (4) 1 (4)
		

**Fig. 1:**
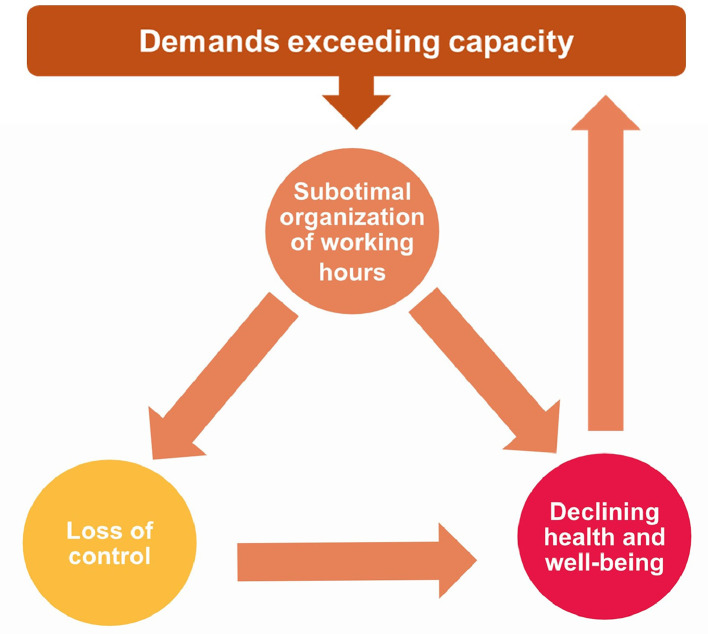
**The three main themes, their relations to each other and the context.** As a result of increased pressure on healthcare organizations during the COVID-19 pandemic, employers flexed their resources by implementing new ways of scheduling working hours. The way working hours were organized was considered suboptimal from the nurses’ perspective and resulted in perceived loss of control and declining health and well-being among nursing staff. Loss of control also contributed to declining health and well-being. Ultimately, this can generate feedback loops which further decreases the capacity of the organization. (For interpretation of the references to colour in this figure legend, the reader is referred to the web version of this article.)

Three main themes with three sub-themes each were identified from the interview data. An overview of the main themes and sub-themes with example quotes is shown in Table 4. Fig. 1 shows how the different themes interrelate, and how they relate to a context where the demands exceed the capacity of healthcare organizations.

**Table 4:** Overview of main themes and sub-themes with example quotes.

**Main themes**	**Sub-themes**	**Example quotes**
1. Suboptimal organization of working hours	1.1 Demanding shifts and shift patterns	*“"I think it's pretty strange to even send out such a schedule, because just looking at it makes you realize that it is going to make someone hit a wall very quickly." – Participant 19*
	1.2 Little room for recovery	*“I didn't get any weekends off with my family and I was supposed to work 4 days [in a row] all the time. I never got any longer periods of leisure” – Participant 6*
	1.3 Poor working hour management	*"They say, 'Well, these are our working hours, so you'll have to look for another job.' And then my competence wasn't worth anything. And that's probably been consistent, that many feel like pawns in a game.” – Participant 1*
2. Loss of control	2.1 Lost influence over working hours	*”Absolutely no influence [over working hours]. Absolutely not. Zero.” – Participant 14*
	2.2 Unpredictability	“*It could happen in the same day, like “can you switch from the day shift to the evening tomorrow?*"*, or that they ask you on a Tuesday if you can work the weekend instead of Thursday and Friday for example”” – Participant 5*
	2.3 Blurred boundaries between work and leisure	*“I went out of the house once to go someplace and realized I had forgotten my phone at home, so I went back to get it in case they would call me in again.” – Participant 18*
3. Declining health and well-being	3.1 Impaired sleep	*”It became more and more clear that I had huge sleep problems. I could not sleep […] I am still imbalanced when it comes to sleep, and I am afraid all the time that my issues will return”. – Participant 20*
	3.2 Mental and physical changes	*“I felt low. Not depressed, but generally low and tired.” – Participant 3*
	3.3 Extreme fatigue	*“I felt fatigued in the sense that I didn't take any initiatives and didn't have the energy to do anything at home. Cleaning felt like it took forever. I didn't see many friends.” - Participant 17*

## 5 Suboptimal organization of working hours

Nurses perceived the organization of working hours during the COVID-19 pandemic as suboptimal. Three sub-themes were identified: demanding shifts and shift patterns, little room for recovery, and poor management of working hours.

### 5.1 Demanding shifts and shift patterns

A reoccurring theme was the perception of shifts and shift patterns as being demanding, e.g. long work weeks (up to 60 h), long shifts (12–14 h), night work, rotating between day/evening/night shifts, short rest time between shifts (9–10 h), double shifts, and overtime. Some nurses described not taking on any extra shifts due to feeling like they couldn't manage working any more than they already did, and some suggested that there should have been a work hour reduction given how demanding the work environment had been. Nurses who worked on the crisis agreement described that the long shifts (12,5 h), long work weeks (48 h), and the number of consecutive shifts (4–5, sometimes more) were perceived particularly demanding, and some expressed that fixed schedules would have been preferred over the crisis agreement.

*“I think it was 4 workdays and 2–3 days off [on the crisis agreement]. And if you think about that it was 12,5 hour shifts… It is quite a long time to work, and it is heavy” - Participant 20*

Nurses who worked on the fixed 4–2 schedules (four workdays followed by two days off) also expressed that the working hours were too demanding despite working shorter shifts than those working on the crisis agreement. Participant 8 stated that working 4–2 schedules felt like *“being on a hamster wheel”* and that participatory working time scheduling would have been a better option*.* At the same time, some nurses who had used participatory working time scheduling during the pandemic described that this also resulted in demanding working hours as schedules were changed by the employer to meet production needs, which in some cases entailed working 5–6 shifts (including night shifts) in a row, up to 48 h per week, short rest times (<11 h) between shifts, working double shifts or many shifts on weekends. Participant 3 talked about this saying *“I believe in flexible schedules, but if you don't get time off, it's not good. In that case, it is better to have fair fixed schedules”.*

The nurses provided some suggestions for how to make the working hours less demanding, which included for example alternating the crisis agreement among nursing staff, having more time off after a work period especially following night shifts nights, and avoiding scheduling morning/day shifts in the beginning of a work period following night work. The nurses also emphasized the need to ensure sufficient staffing levels, as it would allow more people to share the working hours and workload and prevent excessive strain on nursing staff.

### 5.2 Little room for recovery

Overall, it was described that there was little room for recovery from work, and some nurses talked about having only one or maximum two days off in-between every work period, leaving little room for activities other than sleeping and resting.

*“Many thought that it was too little recovery. You finish in the evening, go home, are quite exhausted and don't have time to unwind properly. The first day off you are tired. Then you have another day where you can't really stay up or do anything because the next day you have to be at work again at 7 a.m. “– Participant 6*

Among nurses working on 4–2 schedules, switching from a clockwise rotated schedule (day-day-evening-evening) to a counterclockwise rotation (evening-evening-day-day) was considered positive with respect to recovery as it meant that they got 16 more hours at home between two blocks of shifts. At the same time, this resulted in a quick return, i. e. 9–10 h between shifts, in the middle of each block of shifts, which some described as too little. Also, both the crisis agreement and the 4–2 schedules frequently placed shifts on either a Saturday or Sunday resulting in very few weekends without work. Being imposed to work more weekends limited the room for spending time with family and friends, or for social activities. Some nurses suggested that it would have been more beneficial to alternate weekend work between nursing staff to ensure time off during weekends on a regular basis.

*“I was scheduled to work so many weekends, which made my family life completely messed up […] I was out of sync with my family” – Participant 19*

Another aspect which limited the time for recovery was that, in some cases, nursing staff were not offered any summer vacation. Vacation was described as an important source of recovery, and participant 11 stated *“one would have needed 4 weeks [of summer vacation] for recovery”.* It was suggested that vacation should be prioritized in case of a similar situation in the future, and that employers should attempt to provide fixed periods of recovery (e.g. several days up to a week), where nursing staff are guaranteed to get time off for recovery during a crisis.

### 5.3 Poor management of working hours

The management of working hours during the pandemic was overall perceived as poor. Working hours and new types of schedules were decided by for example upper management who had limited insight in the work situation at the sharp end. At one hospital, an external actor was brought in to assist the scheduling process, but, according to the nurses, these schedules were not well adapted to the healthcare sector and had to be changed shortly after they were finalized. Technical barriers caused further problems in the scheduling process, and some of the issues persisted for a long time. First-line managers, who were responsible for ensuring that working hours were sustainable and safe, had limited time and resources to deal with these matters. The general picture given by the nurses was that managers’ focus had mostly been on covering shifts, and less on the sustainability of working hours when planning schedules.

*“I think that they just thought about covering shifts with the people they have […] It must have been the case given what the schedules looked like” – Participant 3*

The nurses emphasized the importance of managements attentiveness towards employees needs in a situation like the COVID-19 pandemic and suggested that management should listen more to those who express that they are not coping well with working in a certain way (e.g., night work) or who may require individual adjustments of schedules (e.g., due to family situation). Nurses also expressed a need for better preparedness for the future regarding working hours during extreme conditions. Suggestions included having a system in place for working hours and scheduling that could spring into action in the event of a crisis, as opposed to coming up with ad hoc solutions. However, the nurses also described that the management of working hours and scheduling practices improved over time. For example, the working hours became less intense, and more consideration was given to individual prerequisites. In some cases, employers tried to avoid long-term strain on certain individuals by alternating demanding shifts or schedules among nursing staff. Nurses expressed their understanding for the managers’ situation and that planning working hours during the pandemic had likely not been an easy task.

## 6 Loss of control

Nurses described that the organization of working hours resulted in loss of control in terms of lost influence over working hours, unpredictability, and blurred boundaries between work and leisure during the pandemic.

### 6.1 Lost influence over working hours

Nursing staff experienced lost influence over working hours as the organizations went from participatory working time scheduling to fixed schedules or activated the crisis agreement. Thereby nursing staff also lost the possibility to put “veto” on certain days they wished not to work, which could result in having to use vacation to be able to attend private commitments.

*“I can plan my life [with participatory working time scheduling]. I can plan for when I am going away, and I will add my veto there. Then I still have a life in a way” – Participant 9*

The nurses overall emphasized the importance of being able to influence their working hours and participant 1 stated that *“this freedom, that we get to set our own schedule, is very important”.* Fixed schedules, including 4–2 schedules, were sometimes referred to as “forced schedules”, and some of those who worked on these schedules described a strong feeling of frustration with their employer for making scheduling decisions without nursing staff's involvement or input. Some nurses even considered leaving their job due to feeling trampled on. It was expressed that nursing staff should have been included in the decisions around working hours during the pandemic as they were the ones affected by the changes.

### 6.2 Unpredictability

During the pandemic, the working hours were provided on shorter notice than usual, in some cases only 2–4 weeks in advance, and nursing staff were sometimes asked to change their schedules after they had been finalized. The actual hours sometimes varied drastically between weeks (e.g. 50 vs 20 h). Working hours for nurses working on the crisis agreement were particularly associated with unpredictability and sudden scheduling changes. According to participant 16, schedules for staff on the crisis agreement *“could fall apart at any time, in any way”.* Staff shortages also required nursing staff to take extra shifts or work overtime. Overall, nurses experienced an uncertainty regarding how long new schedules would be in action, as the initial timeframes were extended several times due to remaining pressure from the pandemic. Some nurses stated that it would have been beneficial to have a clear timeframe for how long the new working hour schedules would be in action.

*” You didn't know when it was going to end, and if it was going to end. It would have been psychologically important to have it [crisis schedule] over a limited time period” - Participant 17*

However, nurses working on the 4–2 schedules described a high predictability with regard to their schedules, as they rolled continuously in a pattern of four working days followed by two days off and rarely entailed overtime/extra shifts.

### 6.3 Blurred boundaries between work and leisure

The nurses were often reminded of work during free time, as the pandemic was frequently discussed in the media and in society overall. Working on the crisis agreement also required nursing staff to be on-call during free time. Although these nurses stated that they didn't get called in very often, the feeling of always having to be available was considered challenging. Some nurses suggested that on-call duty should have been alternated between employees working on the crisis agreement in order to allow better disconnection from work.

*“If I had known I was free when I was not scheduled to work, I think it would have helped me to relax during free time. To be able to focus on recovery before the next shift. Instead, I had to wait around at home in case they would call” – Participant 18*

Worries about how the pandemic would proceed was described as something that occupied the mind of nurses, especially in the beginning of the pandemic. Some nurses also described that they during free time were thinking about difficult patient cases, how patients were doing, if they as nursing staff had done enough for a patient, or if work tasks had been done right.

## 7 Declining health and well-being

The nurses described different ways in which their health and well-being declined during the pandemic, including impaired sleep, physical/mental changes, and extreme fatigue.

### 7.1 Impaired sleep

Some nurses experienced difficulties falling asleep, as well as shortened and (or) interrupted sleep. Some stated that the initial impact of the pandemic resulted in sleeping problems, while others experienced that their issues developed over time. In some cases, these sleep disturbances became chronic and lasted even after the most intense periods of the pandemic were over.

*“I have always fallen asleep easily […] But after this [working nights during the pandemic] I can't. It is like you have to empty your mind of everything, thinking through the entire night in order to move on and fall asleep. It is like a computer that won't shut off before you make an update” – Participant 16*

Some of the factors contributing to poor sleep, according to the nurses, were having worked stressful shifts, difficulties to unwind and thinking about work while trying to sleep. The nurses also described specific scheduling characteristics that were associated with poor sleep, including quick returns, irregular working hours, night work, rotating between day, evening, night shifts and working several consecutive night shifts. Some nurses who normally worked only day shifts started working nights during the pandemic and suffered sleep problems as a result. Participant 21 for example expressed that a single night shift was manageable, but *“…if I work two or three nights, I know that I will have great difficulties to sleep*”. Some nurses described how their sleep impairment had resulted in having to take sleep medication or, in some cases, sick leave. However, not all nurses had experienced sleep issues during the pandemic, while some had experienced sleep impairment prior to the pandemic as well.

### 7.2 Physical and mental changes

Some of the nurses experienced mental and physical health issues during the pandemic, including high blood pressure, hair loss, weight loss/gain, losing muscle mass, stomach pain and migraines, as well as stress, depressive thoughts, feeling sad or less happy, and feeling burned out. In some cases, stress-related issues persisted over time, and despite getting longer periods of recovery (i.e., during vacation).

*“I had this propeller in my chest with concerns and fears of what was going to happen […] During the third week [of vacation], this propeller in my stomach started spinning. I am going back. I don't want to go back” – Participant 8*

Nurses who experienced worsening health described how they for example had to stop working on the crisis agreement, that they couldn't take it anymore, and some were ultimately put on sick leave. Some nurses also identified deteriorating health among their colleagues. Participant 11 explained that “*we have a lot of sickness absence right now [April 2021]. I think people are tired after COVID. People are exhausted”*. It was described as difficult to see that colleagues were affected negatively by the work situation.

### 7.3 Extreme fatigue

Some nurses described a feeling of extreme fatigue both physically and mentally, which manifested itself in terms of for example feeling intensely tired even after working just one shift or feeling indifferent with neither energy nor motivation to for example play with one's children or engaging in friends and loved ones.

*“Small things require longer recovery now than they did before” – Participant 18*

Some nurses described how almost all leisure time was used for recovering from work in terms of sleeping or resting, and how there was very little energy left to engage in activities such as social activities or physical exercise. The fatigue experience consisted of cognitive difficulties (e.g., poor memory, poor concentration, feeling confused), declining enthusiasm towards work, and feeling tense, irritable, sad, slow or dull. The nurses attributed their fatigue to extensive working hours and work environmental demands, as well as not getting sufficient sleep, worrying and constantly thinking about work. The nurses described feeling most fatigued during leisure time. Participant 6 talked about how they would *“run out of steam”* when coming home after a shift, while participant 9 described *“walking around in a haze”* during free time.

## 8 Discussion

The findings from the present study shed light on how some of the adaptive strategies used by healthcare organizations, such as flexing organizational resources (reorganizing working hours) in order to adapt to escalating demands during the COVID-19 pandemic, contributed to declining health and well-being among nursing staff. Nurses described insufficient recovery in relation to the demanding working conditions, in some cases leading to extreme fatigue and cognitive, emotional and physical health problems. In turn, these could jeopardize patient safety by increasing fatigue related risks (Lerman et al., 2012) and lead to loss of human resources through increasing sick leave or turnover (Dall'Ora et al., 2018; Poon et al., 2022). In other words, the results demonstrate how short-term adaptive organizational strategies can have a wider system impact by creating feedback loops, which could be draining the human capital within the organization in the long run (Page et al., 2023; Hernandez et al., in press). Below follows a discussion of how different aspects of working hours, and factors such as control and recovery may be related to health and safety consequences in a wider system perspective.

Previous research has highlighted that demanding working hour characteristics, similar to what the nurses in this study experienced (e.g. long working hours, frequent night work), constitute an increased risk for negative health and safety consequences (Wagstaff and Lie, 2011; Wong et al., 2019). The nurses in the present study also described decreased room for recovery, which might be another important mechanism for health and safety (Geurts and Sonnentag, 2006; Kecklund and Axelsson, 2016). Thus, there is a need to identify ways to make working hours less demanding and enable sufficient recovery, especially during periods of increased pressure. This is also in line with the nurses’ suggestions for the future, which included alternating employees working on demanding schedules, more time off between two blocks of shifts, and having a clear plan for working hours in case of a crisis as it would decrease the need for ad hoc solutions. Nurses also pointed out the need to ensure sufficient staffing levels, which is in line with previous findings showing that staff shortages and lack of certain competences were among the challenges faced by healthcare organizations during the COVID-19 pandemic (Hadian et al., 2022; Jabbari et al., 2022; Hernandez et al., in press). Taken together, this implies that healthcare organizations may need to increase their efforts to improve staffing levels and skill mix, as well as to establish well-defined guidelines for managing working hours and recovery during periods of increased pressure.

There were differences among nurses when it came to how working hours were scheduled during the pandemic. In line with previous findings, nurses working 12,5 h shifts and 48 h work weeks on the crisis agreement experienced poor health outcomes (Hoedl et al., 2021; Lang et al., 2020; Sagherian et al., 2023). On the other hand, nurses who worked on 4–2 schedules were unique in describing a consistent pattern of workdays and days off, shorter shifts and shorter work weeks, which can be considered a positive aspect of these schedules. However, nurses working on 4–2 schedules also expressed that their working hours were suboptimal and some experienced poor health outcomes, which was an unexpected finding. One major reason given for the negative evaluations of 4–2 schedules was that compared to before the pandemic, shifts were more likely to occur on weekends, thereby reducing the time available to spend with family and friends. Social support has been described as an important factor for managing work-related stressors among nurses during the pandemic, where family members were considered to provide a particularly important emotional support (Goh et al., 2021), suggesting that there could be a deeper dimension to why weekend work and other unsocial aspects of working hours were considered problematic by the nurses. Thus, despite efforts to avoid extensive working hours at the organizational level, it appears that nurses were still negatively affected by their working hours during the pandemic, highlighting the importance of taking recovery and social aspects into consideration when designing work hour schedules.

Other negative aspects mentioned by nurses included lost influence over working hours, that schedules were implemented without involvement or input from employees, a challenging work environment and absence of longer recovery periods. These further highlights that the number of working hours is likely not a sole predictor of poor health among nurses during the pandemic, and that other factors must be considered. Employee influence over working hours (for example through participatory working time scheduling), which in many cases was removed during the pandemic, can serve a protective purpose against negative consequences of demanding and unpredictable working hours, for example by improving work-life balance (Ala-Mursula et al., 2005; Henly and Lambert, 2014; Nijp et al., 2012). It could also constitute an important resource in terms of control to balance the increasing demands (demanding working hours, high workload). Losing influence could thus have further impacts including increased strain, depletion of energy and ultimately, poor health (Bakker and Demerouti, 2007). This provides further context as to why some nurses experienced negative effects despite not working extensive hours during the pandemic.

Some nurses stated that they would have preferred participatory working time scheduling during the pandemic. However, our findings do not necessarily point towards that participatory working time scheduling is the best option during a crisis. Firstly, it was described by some that such schedules resulted in changes on short notice, and that overtime, double shifts and short recovery periods were frequent. Moreover, previous research has highlighted that under normal circumstances, participatory working time scheduling is a time consuming and complex process, which can be facilitated by an active leadership (Epstein et al., 2023). During the pandemic, the managers were facing high job demands according to the nurses, which might imply poor preconditions for participatory working time scheduling. Thus, achieving sustainable schedules using these schedules may be challenging during a crisis. Further research is therefore needed to establish ways to optimally balance employee influence over working hours, which appears to constitute an important resource at the individual level, against the need for more organizational-level control over working hours during a crisis.

When it comes to recovery, one aspect that disrupted recovery among the nurses during the pandemic was the feeling of blurred boundaries between work and leisure, exemplified by constantly being on-call, and thinking about work during free-time. This could hinder the ability to psychologically detach from work and prolong physiological stress-responses outside the work setting (Agolli and Holtz, 2023; Brosschot et al., 2018). Psychological detachment from work (i.e. the ability to “let go” of thoughts and feelings related to work during free time) has been considered an important recovery mechanism and can serve as a mediator between job demands and impaired health (Agolli and Holtz, 2023; de Jonge et al., 2012). Detachment may however be hindered when job stressors are high, contributing to a recovery paradox at the individual level (Sonnentag, 2018). Both poor recovery and poor detachment have been associated with unfavorable health consequences and negative work-related outcomes such as poor task performance (Sonnentag et al., 2022; Wendsche and Lohmann-Haislah, 2017). On the other hand, detachment from work can be facilitated by recovery activities outside of work including social, physical, and low-effort activities (Agolli and Holtz, 2023; Sonnentag, 2001). However, the nurses described experiencing limited engagement in leisure activities during the pandemic due to that the demanding working hours left little room for such activities and due to experiencing extreme fatigue. Previous research has highlighted that work-related communication, being available outside work, and high workload can hinder detachment (Agolli and Holtz, 2023), which is in line with the nurses’ descriptions of their work situation during the pandemic. It was suggested that this could be improved by alternating on-call work between employees. Thus, it may be important for healthcare organizations to consider ways to alleviate employees from being on-call over extensive periods of time and to, as previously mentioned, take social aspects into account when scheduling working hours. Further research is needed to establish how to promote detachment from work and to counteract the recovery paradox at the individual level under extraordinary working conditions, such as during the COVID-19 pandemic.

Our findings also show that extreme fatigue resulted in an increased need for recovery. At the same time, organizations may have a limited capability to meet this need when the pressure is high, possibly resulting in a downward spiral of having to meet high demands at the organizational level, leading to increased fatigue and need for recovery at the individual level. Fatigue constitutes a risk for impaired workplace safety and poor health outcomes (Lerman et al., 2012; Knoop et al., 2021). Thus, entering a downward spiral of high demands and poor recovery may create negative feedback loops where health decline, increased fatigue and poor performance at individual level jeopardize safety and further impacts the capacity and performance of the organization, putting organizations in the state of an organizational recovery paradox (Hernandez et al., in press). Loss of control may contribute to these feedback loops trough dissatisfaction (as described by the nurses) and the loss of a potentially important buffer against high job demands, further increasing the risk of energy depletion and poor health (Ala-Mursula et al., 2005; Bakker and Demerouti, 2007).

In sum, our results suggest that flexing resources can result in 1) suboptimal organization of working hours including demanding working hours and insufficient recovery and 2) loss of important resources for employees, such as control and support (i.e., social support during leisure), as well as blurred boundaries between work and leisure which hinders detachment from work. This can lead to secondary outcomes including increasing levels of fatigue, poor sleep, and declining health/well-being at the individual level. In turn, these secondary outcomes can contribute to feedback loops and ultimately deplete human resources, decrease the capacity and performance of organizations, and increase strain over time.

### 8.1 Methodological considerations and limitations

While the coding process can be susceptible to errors due to a lack of persistence and personal preconceptions, the authors diligently examined and deliberated on the accuracy of coding throughout the analysis process. The involvement of all authors in each step of the analysis process minimized the risk of biased interpretations of data and enabled researcher triangulation, thus enhancing study credibility (Nowell et al., 2017). The research findings were well documented and described in detail, including the different steps of the analysis process which followed a clearly defined methodological approach (Braun and Clarke, 2006, 2022). This enhanced the dependability of the study findings. Generalizability is constrained due to the use of self-selected sample and the qualitative nature of the study. However, the well-defined context of the sample, representing various work organizations and health care settings as well as a variation in age and years of experience amongst participants, provide valuable insights into the perspectives of nurses working during the pandemic. Thus, the study findings may be transferrable to similar contexts and situations. Confirmability was obtained by going back to the data transcripts in the final stages of the analysis process to check findings against the raw data, ensuring that themes and sub-themes reflected the answers given by the respondents. Furthermore, data extracts were presented in tables and text, demonstrating the direct link between raw data and the findings presented (Nowell et al., 2017). All authors were female and had prior experience from working with qualitative research within the healthcare context. The authors were not affiliated with the employer organizations, which minimized pressure for socially acceptable and biased answers. Strengths of the study lies in the diverse work contexts and varied backgrounds of the participants, which captured a variety of experiences and enriched the exploration of working hours, recovery, sleep and health/well-being.

However, a limitation of the study is the absence of perspectives from individuals who chose not to be interviewed or who were not invited to study participation (e.g. employees in other regions/hospitals). It is therefore not possible to rule out that the inclusion of individuals from other contexts could have contributed with additional information. We also recognize the potential challenges for some disadvantaged groups, such as the extremely tired, distressed or those in ill-health, to participate. Thus, there is a possibility that the views expressed by the nurses who consented to the study may not represent a comprehensive spectrum of opinions. Nevertheless, the study revealed insightful personal narratives about how working hours impacted the lives and health of the nurses. Data collection occurred at different timepoints during the pandemic, introducing variations in the time spent working under challenging conditions. This may have influenced nurses’ perceptions of the present topics with memories and involvement in pandemic-related issues reported months later.

## 9 Conclusion

Increased pressure on the Swedish healthcare system during the COVID-19 pandemic resulted in suboptimal organization of working hours and loss of control, which had negative consequences including insufficient recovery, fatigue and declining health among nursing staff, potentially leading to wider system impact through negative feedback loops. Based on our results, in order to minimize negative consequences in relation to working hours and recovery in the event of future crises, we suggest the following;

*Ensuring sustainable working hours* that can be applied over longer periods of time to maintain health and safety in the event of a future crisis. Further research is needed to determine how to schedule sustainable working hours during a crisis, but according to our findings, scheduling elements such as having >1–2 days off between work periods, frequently scheduling longer periods of recovery, alternating weekend work, and having several days off when switching between day- and night work may be particularly important to consider.

*Allowing nursing staff some level of influence over working hours* as it can constitute an important resource at the individual level and serve as a mean to minimize work-life conflict and avoid dissatisfaction. How to involve employees in creating schedules that can spring into action in crises situations should be explored in future research. This could limit the need for ad hoc solutions and conflicts in the event of a future crisis.

*Promoting psychological detachment from work* may be crucial during a crisis to enhance recovery, and how this best can be supported during a crisis should be further addressed in future research. Based on our findings, alternating on-call responsibilities between employees may be a way to tackle this issue. Scheduling weekend work is another aspect to consider in order to enable recovery activities outside work and support from family and friends.

### Funding sources

This research was supported by a research grant from 10.13039/501100002706Afa Försäkring (grant nr. 200204). The research was conducted without any involvement from the funder.

## CRediT authorship contribution statement

**Isabelle Hernandez:** Writing – original draft, Investigation, Formal analysis, Conceptualization. **Marie Söderström:** Writing – review & editing, Methodology, Investigation, Funding acquisition, Formal analysis, Conceptualization. **Ann Rudman:** Writing – review & editing, Methodology, Formal analysis, Conceptualization. **Anna Dahlgren:** Writing – review & editing, Supervision, Methodology, Investigation, Funding acquisition, Formal analysis, Conceptualization.

## Declaration of competing interest

The authors declare that they have no known competing financial interests or personal relationships that could have appeared to influence the work reported in this paper.
